# Cases of Diseased Bladder and Testicle. Illustrated with Etchings

**Published:** 1820-04-01

**Authors:** 


I. *Cases of diseased Bladder and Testicle. Illustrated with Etchings.* By WILLIAM WADD, Surgeon. 4to. p. 72, and 21 plates. 1815.

---

II. *Cases of diseased Prepuce and Scrotum. Illustrated with Etchings.* By WILLIAM WADD, Esq. Surgeon Extraordinary to His Royal Highness the Prince Regent. 4to. p. 31, and 10 plates. 1817.

---

III. *On the Malformations and Diseases of the Head. Illustrated with Etchings.* By WILLIAM WADD, Esq. F.L.S Surgeon Extraordinary to His Royal Highness the Prince Regent. 4to. p. 21, and 11 plates. 1819.

THE press, which might be considered as conferring a kind of immortality on Books, and consequently on the memories of their Authors, is, in reality, a most potent engine of destruction to both. A moment's reflection will solve this seeming paradox. The press multiplies, with almost incredible speed, the number of books, and the facility of competition. When books were in manuscript, they were few — read by few — and it did not require half a life-time to peruse them; they were, therefore, read often, and remembered. They are now multiplied to such an extent, that, to use the words of an able critic, " if we continue to write, at the present rate, for two hundred years longer, there must be some new art of *short-hand reading* invented, or all reading will be given up in despair." The consequence at present is, that each generation eclipses the writings of its predecessor; and, in a progressive science like ours, the moment an author drops into the grave, his work is amalgamated with the existing mass of information, and himself soon forgotten.

An analytical journal is calculated, in some degree at least, to protract this appaling annihilation of our embodied thoughts, and literary existence, by forming a kind of *Selectæ e Medicis,* in a shape and size that enable it to circulate far and wide, and render it easy of access and perusal. " When we look back, says an ingenious anonymous writer, " upon the havoc which two hundred years have made in the ranks of our immortals; and above all, when we refer their rapid disappearance to the quick succession of new competitors, and the accumulation of more good works than there is time to peruse, we cannot help being dismayed at the prospect which lies before the writers of the present day. By such a work as the present, however, [alluding to Campbell's Specimens of British Poetry] this injustice of fortune may be partly redressed; some small fragments of an immortal strain may still be rescued from oblivion; and a wreck of a name preserved which Time appeared ready to swallow up for ever."*

If this be true of Poetry, which has not been improved upon since the days of Homer, how much more applicable is it to Medical Science, where a *system* that required a whole life to erect, is too often rent asunder, like a temple by lightning, as soon as the test of experience is applied! It is useless then to be too anxious about our posthumous fame, which may never reach our ears; our business is with the present time, which fleets speedily away, and which it is our duty as well as interest to improve.

The three volumes which lie before us have not, probably, been circulated in proportion to their author's and their own deserts; partly, perhaps, from the roughness and homeliness of the plates, which may not be sufficiently attractive for the fastidious eye of the present critical generation.

Mr. Wadd is entitled to the praise of great industry and zealous observation, in recording the ravages of disease on the living structure, by means of cheap etchings and short histories of the complaints;—but as the letter-press is principally illustrative of the plates, and the plates of the letter-press, the work becomes incapable of regular analysis; and all we can be expected to do, in such a case, is, to exhibit a few specimens of the letter-press, and also one of the plates, which, as far as they go, may be useful matter in our Journal, and sufficiently indicative of the nature of the work reviewed.

The first volume is occupied with affections of the urinary organs, the complicated structure of which, renders them very liable to disease. Mr. Wadd, in the opening of the work, expresses his astonishment that the pupil and successor of John Hunter should have considered a diseased enlargement as a natural formation of the prostate gland—alluding to the supposed discovery of a middle lobe. We shall select the subject of the 5th Plate for our first specimen of the verbal descriptions.

---

* Edinburgh Review, March, 1819.

*Case of Enlargement of the Prostate Gland, and thickened Bladder, the Surface of which was covered with Coagula.*

"The patient was sixty-three years of age, and had been nearly twenty years afflicted with difficulty in making water. He applied to me in 1804, under a supposition that his complaints arose from strictures; the examination of the case, however, proved the seat of the disease was wholly confined to the bladder and prostate. He was by trade a stone-mason; but had not been able to work for several months, owing to his infirm state. On hearing his history, it appeared that his miseries had been much aggravated by large doses of gin and water, which a brother mason had recommended to "force his water;" a practice in daily use among the labouring class; and when they have once taken to it, as a facetious author observes, it is no uncommon thing for them to like the disease for the sake of the remedy. A more deplorable condition can scarcely be conceived. He had a constant irritation at the neck of the bladder, accompanied with a burning pain in the glans penis, a frequent discharge of urine, which sometimes flowed involuntarily by day, and constantly during the night. Hence the skin of the scrotum was so much inflamed as to render the least motion difficult and painful, and he was very feverish, with head-ach, and his stomach rejected food.

"The urgency of his symptoms being relieved by means of fomentations, the warm bath, anodyne clysters, &c. the case appeared likely to be served by the vesicæ lotura. Though the prostate gland was evidently enlarged, the catheter could be passed, with some difficulty. The first time I injected the bladder, it would not admit more than half an ounce of warm water, which was almost instantly expelled. The next attempt, two days afterwards, was attended with no better success, and by a daily repetition, at the end of a fortnight, nearly an ounce remained for five minutes. By this time, and by the means mentioned above, the general irritation had abated, and the state of health was improved; but the irritability of the bladder being still very distressing, the injection was persisted in every third day. After pursuing this plan, in addition to general remedies, for six weeks, he was so much better, as to commence his usual occupation; and soon ceased to require my assistance.

"I saw nothing of him for nearly two years, when he came again with the parts in as miserable a state as before, and the constitution greatly impaired. He had returned to his favourite remedy, and now had the odour, and jaundiced appearance of a dram-drinker. The former means were resorted to, with some temporary relief; but the vesicæ lotura could not be repeated, as he could not bear the catheter to be passed. After a few weeks he died." 13, 14, 15.

A sufficiently expressive etching of this deplorable case, is given in elucidation.

Mr. Wadd observes, that since 1807, he has seen so many instances of relief afforded by the *vesicæ lotura*, that he is surprized it is not more frequently resorted to by practitioners.

Towards the close of the first volume, there are many valuable cases and drawings of diseases of the testicle, and judicious remarks on hydrocele.

We have here the pleasure of introducing a specimen of Mr. Wadd's etchings, with its corresponding explanation.*

" PLATE XX.

" These preparations are selected from many others, as they represent an appearance of the testicle in hernia congenita not unusual, but I have thought not sufficiently noticed.

" The subject of the first, at about forty years of age, was killed by falling from the roof of a house, and in examining the body, this rupture was discovered. In this kind of hernia, adhesions are particularly numerous, and usually elongated; which is easily accounted for when we reflect that the testicle, in its passage, is not to be considered as moving loosely between the intestines, but every where attached to those parts with which it is in contact. This readily explains that appearance of the intestine adhering to the the testicle, in some places, by firm fibrous bands, such as are seen in the upper part of the sac, spreading over the spermatic chord.

" The frequent occurrence of these adhesions explains also the difficulty so frequently met with in reducing such herniæ; and that very difficulty should always remind the surgeon of the probability of such a cause." *Vol. I. p.* 67.

The second volume, on Diseases of the Prepuce and Scrotum, also contains a series of interesting cases, plates, and remarks, particularly on phymosis and paraphymosis, which are deserving of the surgeon's attention.

In the third, or recent volume, which is the only legitimate object of notice in this Journal, (being published within its æra) the author aims at the illustration of some curious facts in the pathology of the brain, and endeavours to connect morbid alterations of structure with some previous symptoms.

The following case is taken at random.

" PLATE IX. exhibits a cancerous affection of the eye-lids and surrounding parts, accompanied with diseased state of the brain, the effect of external injury.

" This man, about fifty years of age, received a violent blow on the eye, from the end of an oar. Great inflammation ensued, which subsided without any injury to his sight, though he was

* See the Plate in front of this Number.

never free from pain in his head. Six months afterwards, an insect stung him in the same eye, which instantly caused great swelling, and destroyed the power of vision. Inflammation and pain were excessive, and in the course of a few weeks the eye sloughed away, leaving the orbit bare, and the eye-lids as represented in the drawing. He lingered some months in misery, and retained his faculties to the last.

"Upon the dissection of his head, the right hemisphere of the brain was found covered with pus, and a considerable portion of the anterior lobe in a state of suppuration, but chiefly in a line parallel with the foramen opticum. The brain adhered firmly to the dura mater, covering the orbitary process of the frontal bone, and extending to the os petrosum.

"On opening the ventricles, a small quantity of fluid was found, of a yellow colour, and fœtid smell. The thalami nervorum opticorum, were of a dark purple colour.

"The brain and cerebellum were unusually soft, and the veins on the surface so pale, as in some parts to be scarcely visible.

"Part of the orbitary process was destroyed, making an opening large enough to admit the passage of two fingers.

"In a similar case, of a patient of the late Mr. Bureau's, who lost the use of one eye from a blow on the forehead, abscess was formed on the anterior lobe of the brain; the orbitary process denuded, and a portion of it, near the foramen opticum, in a state of ulceration. This man also was sensible to the last; and his case, with the two preceding, are given, as illustrative of the extensive injury the brain may sustain, without affecting the faculties." 18.

We consider Mr. Wadd's endeavour to convey to the public, in a cheap and plain manner, the results of his pathological experience, as very deserving of encouragement; and we think it is a duty incumbent on medical associations and book clubs, at least, to extend their patronage to such laudable undertakings.

We understand that the work is to be followed up by Miscellaneous Cases in Surgery. We wish every success to its able and indefatigable author.

